# The Effects of Toxic Heavy Metals Lead, Cadmium and Copper on the Epidemiology of Male and Female Infertility

**DOI:** 10.5935/1518-0557.20220013

**Published:** 2022

**Authors:** Aliasghar Manouchehri, Samira Shokri, Mohadeseh Pirhadi, Mohammad Karimi, Saber Abbaszadeh, Ghazal Mirzaei, Mahmoud Bahmani

**Affiliations:** 1 Assistant Professor, Department of Internal Medicine, Shahid Beheshti Hospital, Babol University of Medical Sciences, Babol, Iran; 1 Department of Environmental Health Engineering, Division of Food Safety & Hygiene, School of Public Health, Tehran University of Medical Sciences, Tehran, Iran; 3 Department of Infectious Disease, Imam Reza Hospital, Amol Mazandaran, Iran; 4 Department of Biochemistry and Genetics, School of Medicine, Lorestan University of Medical Sciences, Khorramabad, Iran; 5 Biotechnology and Medicinal Plants Research Center, Ilam University of Medical Sciences, Ilam, Iran

**Keywords:** epidemiology, infertility, heavy metals, women, men

## Abstract

Infertility is a major problem in modern society that affects a significant number of couples around the world. Heavy metals and a number of other factors have been causally linked to infertility. The aim of this study was to determine the effect of heavy metals lead, cadmium, and copper on the epidemiology of male and female infertility. Searches for articles published from 1982 to 2020 using related keywords such as male and female infertility and heavy metals were performed in scientific databases PubMed, Google Scholar, Science Direct, and others. The results showed that, in recent years, the number of infertile individuals has increased. Various environmental, occupational, and genetic factors have been described as potential causes. Heavy metals lead, cadmium, and copper cause infertility in couples through various mechanisms, such as changes in sperm motility factors, decreased semen quality, or effects on the egg. Exposure to physical phenomena such as radiation (ionized or microwave) and heat; stress and mental disorders; chemicals from cigarettes, respiratory pollutants (lead), insecticides and pesticides; anesthetic gases; and mercury and cytotoxic drugs may also contribute to the onset of infertility.

## INTRODUCTION

Lifestyle changes arising from industrialization have affected the health of men and women. According to the World Health Organization (WHO), reproductive health is affected by a number of factors that may potentially lead to infertility (Sarvari *et al*., 2010). Infertility is a growing public health problem in most countries, with cases increasing by 50% since 1955. The impact of infertility on the economic and social development of societies is felt at a micro and macro level (Haslegrave & Olatunbosun, 2003; Shafi *et al*., 2016; Direkvand-Moghaddam *et al*., 2014).

Epidemiological studies show that the average prevalence of infertility in the world is 10%. The lowest and highest prevalence of infertility are found in Australia and Africa, respectively (Direkvand Moghaddam *et al*., 2016). The prevalence of infertility in the United States (Marchlewicz *et al*., 2007) is 10-15%; in China (Che & Cleland, 2002), it is around 9%; and in Iran, 13.2% (Zarif Golbar Yazdi *et al*., 2020). Studies show that one sixth of couples suffer from infertility and describe environmental, occupational, and especially genetic factors, as elements associated with its occurrence (Irvine, 1998).

Environmental pollution and exposure to heavy metals are growing problems in the world. Heavy metals alter several reproductive functions in men and women and lead to decreased sperm count, motility, viability, and spermatogenesis, hormonal imbalance, follicular atresia, and delayed egg maturation, to name a few. Heavy metals constitute an important toxicological aspect of fertility. Some metals are called reproductive toxins and are known as “endocrine disruptors (EDCs)”. Exposure to some of these toxic metals is unavoidable (Clementi *et al*., 2008). Cadmium and lead are among the metals that have been the most studied for changing hormone levels and causing fertility problems (Ghahremanei & Ghaem, 2005). Bellas *et al*. (2001) showed that about 5% of the cases of infertility are caused by the harmful effects of exposure to ultra-high-frequency radiation, heavy metals, and herbicides and insecticides in farms. Some occupations, such as working in radiology, industrial and chemical solvent factories, refineries, and lead mines may also interfere with a person’s fertility status (Koskimies *et al*., 2010; Wegner *et al*., 2010). Air pollutants such as exhaust fumes from cars cause defects in the gametogenesis of men and women (Carré *et al*., 2017).

Cadmium and copper can have destructive effects on the reproductive system and lead to infertility (Asadi *et al*., 2013). A study showed that exposure to mercury and lead can disrupt normal activity and impair sperm motility (Bellas *et al*., 2001). Animal studies have described the effects of different concentrations of heavy metals on the motility and ultramorphological parameters of fish sperm, which even in low concentrations may affect the movement of sperm. Lead reduces the weight of the pituitary gland, consequently leading to decreases in the concentration of LH and FSH. LH has a direct effect on the changes in hormones estrogen and progesterone. Decreases in LH cause decreases in the concentrations of estrogen and progesterone (Parvizi & Ellendorff, 1982).

The advancement of science, industrial development, the introduction of products made with these chemicals, and lifestyle changes have increased the number of cases of male and female infertility, with psychological and social consequences. This study aimed to investigate the effects of toxic heavy metals lead, cadmium, and copper on male and female infertility.

## MATERIAL AND METHODS

This study was conducted as a review of articles published between 1982 and 2021. For this purpose, searches for articles were performed on Magiran, Google Scholar, SID, Scopus, PubMed, Science Direct, and ISI databases based on keywords infertility, heavy metals, lead, cadmium, copper, men, and women.

## RESULTS AND DISCUSSION

Although the industrialization of the world in the last century has led to the production of products that in many ways have improved human living conditions, in some cases these products or the jobs created to produce them are harmful to public health and lead to disorders that occur in various organs of the human body, including the reproductive system, which is more sensitive than other organs. Heavy metals can enter the human body through the airways, water, and food. Cadmium is one of the most widely distributed metals in the environment. This metal hurts various tissues of the body, including the kidneys and liver. Cadmium is in the preliminary list of toxic substances and ranks seventh among high-risk toxic substances linked to disease. In the human body, it causes serious damage to the reproductive organs in adults, including the ovaries and testicles. Cadmium causes adverse effects on the body’s organs through two mechanisms. Cadmium replaces zinc in many enzymes and, on the other hand, by reacting with thiol groups of proteins, it changes their structure and function, thus exerting toxic effects (Haji Ghasemkhan, 2007).

Cadmium as a factor in infertility was studied in 60 male volunteers from infertile couples in Nigeria. Researchers have suggested that the strong destructive effect of cadmium on spermatogenesis may be due to systemic cytotoxicity (Akinloye *et al*., 2006). Hemmati Borujeni *et al*. (2020) showed that cadmium chloride treatment in male rats led to the destruction and reduction of germ cells in spermatozoa and infertility tubes. With increasing Cd accumulation in oocytes in female rats, the number of oocytes that reached metaphase II decreased significantly (Písa *et al*., 1990). A study about the effect of heavy metals on camel fertility showed a positive correlation between plasma concentrations of magnesium, and cadmium and percent sperm abnormalities (Meligy *et al*., 2019). Cadmium damages sperm tubes and causes cell degeneration. Studies have described tumors caused by exposure to heavy metals through smoking and occupational hazard. A typical cigarette contains 1.5 µ/gr of cadmium (Chia *et al*., 1994) and causes asthenozoospermia in smokers (Omu *et al*., 1995).

The health of sperm DNA is one of the key factors in fertilization and fertility. Sperm DNA may be damaged by endogenous and exogenous factors and endanger the health of the fetus (Simon *et al*., 2013). Cadmium exposure can increase testicular oxidative stress and ROS production in the epididymis, reducing sperm production, motility, antioxidant capacity, and increasing lipid peroxidation (Marchlewicz *et al*., 2007). Subcutaneous injection of 1 or 5 mg of cadmium chloride (CdCl_2_) into adult male rats results in significant decreases in testicular weight, azoospermia, oligospermia, abdominal prostate, and seminal vesicles (Saksena *et al*., 1977).

Lead extensively damages the nervous system, kidneys, lungs, bone marrow, and causes impaired hemoglobin biosynthesis and anemia, hypertension, miscarriage, preterm birth, male infertility, and learning disorders (Shimbo *et al*., 2001). Lead is considered an environmental pollutant. Therefore, the mechanism of action of lead is such that it can disrupt the endothelial systems of the cell by binding to calcium. Exposure to lead (40g/dl or 25g/dl for years) decreases fertility and increases the risk of miscarriage and stunted fetal growth (preterm delivery, low birth weight) (Bellinger, 2005).

Male infertility for unknown reasons may also be ascribed to environmental and occupational exposure to various chemical agents. Individuals with significant exposure to lead are more likely to show adverse effects on ovarian and testicular growth on account of the effects of exposure on the pituitary and thyroid hormones (Sokol, 1987). Cases of lead poisoning are often reported to affect workers (Khan *et al*., 1995). Kalantari *et al*. (2009) found that the lead levels in people working in the furnace area of a smelting plant were higher than the levels seen in workers from other areas, and that they also had higher levels of prolactin and progesterone than the workers assigned to other parts of the plant. Sharma *et al*. (2013) evaluated the relative effects of lead during pregnancy and postpartum. Lead acetate can cause severe damage to ovarian development during fetal and neonatal life, causing changes in the primary and secondary follicles and impairing production system function (Sharma *et al*., 2013). Omari *et al*. (2015) investigated the number of heavy metals present in cigarette smoke from brands sold in Kenya and found that lead had the highest concentration in all cigarette brands. Examination of experimental data from epidemiological and animal research shows that lead at different concentrations impairs spermatogenesis function, sperm function parameters, and reproductive hormones (Teijón *et al*., 2006).

Chowdhuri *et al*. (2001) showed that lead significantly reduces the ability of sperm to attach to eggs. Lead contamination reduced sperm motility and viability in mice (Golshan-Iranpour & Emami, 2011). Severe toxic effects of mercury on rat sperm have also been reported (Pacey *et al*., 1994). Copper is one of the essential elements needed by the human body. This element is stored in the liver and acts as a catalyst in the chemical interactions involved in making hemoglobin and delivering oxygen to the body’s tissues (Preston & Snell, 2001; Babaei *et al*., 2012). The amount of copper in the semen plasma of infertile men is significantly higher than that of fertile individuals. Copper may mediate the effects of oxidative damage and play an important role in spermatogenesis and male infertility. In concentrations above 100mg/l, copper affected sperm motility in *Dicentrarchus labrax* fish (Kime *et al*., 1996).

## CONCLUSION

Different types of environmental risk factors can lead to disorders in various organs of the human body. Some environmental factors may cause the production of concentrated sperm in men. Exposure to lead, other heavy metals, and pesticides has been linked to male and female infertility. Controversy still looms over the relationship between other factors, such as overexposure to heat, microwave radiation, ultrasound, and other health risks, and infertility.

## References

[r1] Akinloye O, Arowojolu AO, Shittu OB, Anetor JI (2006). Cadmium toxicity: a possible cause of male infertility in
Nigeria. Reprod Biol.

[r2] Asadi MH, Zafari F, Sarveazad A, Abbasi M, Safa M, Koruji M, Yari A, Alizadeh Miran R (2013). Saffron improves epididymal sperm parameters in rats exposed to
cadmium. Nephrourol Mon.

[r3] Babaei H, Kheirandish R, Ebrahimi L (2012). The effects of copper toxicity on histopathological and
morphometrical changes of the rat testes. Asian Pac J Trop Biomed.

[r4] Bellas J, Vázquez E, Beiras R (2001). Toxicity of Hg, Cu, Cd, and Cr on early developmental stages of
Ciona intestinalis (Chordata, Ascidiacea) with potential application in
marine water quality assessment. Water Res.

[r5] Bellinger DC (2005). Teratogen update: lead and pregnancy. Birth Defects Res A Clin Mol Teratol.

[r6] Carré J, Gatimel N, Moreau J, Parinaud J, Léandri R (2017). Does air pollution play a role in infertility?: a systematic
review. Environ Health.

[r7] Che Y, Cleland J (2002). Infertility in Shanghai: prevalence, treatment seeking and
impact. J Obstet Gynaecol.

[r8] Chia SE, Xu B, Ong CN, Tsakok FM, Lee ST (1994). Effect of cadmium and cigarette smoking on human semen
quality. Int J Fertil Menopausal Stud.

[r9] Chowdhuri DK, Narayan R, Saxena DK. (2001). Effect of lead and chromium on nucleic acid and protein synthesis
during sperm-zona binding in mice. Toxicol In Vitro.

[r10] Clementi M, Tiboni GM, Causin R, La Rocca C, Maranghi F, Raffagnato F, Tenconi R (2008). Pesticides and fertility: an epidemiological study in Northeast
Italy and review of the literature. Reprod Toxicol.

[r11] Direkvand-Moghaddam A, Delpisheh A, Sayehmiri K (2014). The Prevalence of Infertility in Iran, A Systematic
Review. Iran J Obstet Gynecol Fertil.

[r12] Direkvand Moghaddam A, Delpisheh A, Sayehmiri K (2016). An Investigation of the Worldwide Prevalence of Infertility as a
Systematic Review. Qom Univ Med Sci J.

[r13] Ghahremanei F, Ghaem H (2005). The effective factors on men infertility: a case - control
study. J Gorgan Univ Med Sci.

[r14] Golshan-Iranpour F, Emami E (2011). The effects of lead on motility, viability and DNA denaturation
of cauda epididymal spermatozoa of mouse. J Shahrekord Univ Med Sci.

[r15] Haji Ghasemkhan A (2007). Industrial Toxicology.

[r16] Haslegrave M, Olatunbosun O (2003). Incorporating sexual and reproductive health care in the medical
curriculum in developing countries. Reprod Health Matters.

[r17] Hemmati Borujeni N, Eidi A, Oryan S, Mortazavi P (2020). Effect of Tilia platyphyllos on Cadmium Chloride-Induced
Testicular Damage in Adult Male Wistar Rats. Res Med.

[r18] Irvine DS (1998). Epidemiology and aetiology of male infertility. Hum Reprod.

[r19] Kalantari S, Khoshi AH, Mohebbi RM, Fooladsaz K (2009). Investigation of blood lead levels and its toxicity in workers of
zinc melting factory of Dandi, Zanjan, Iran. J Adv Med Biomed Res.

[r20] Khan MH, Khan I, Shah SH, Rashid Q (1995). Lead poisoning--a hazard of traffic and industries in
Pakistan. J Environ Pathol Toxicol Oncol.

[r21] Kime DE, Ebrahimi M, Nysten K, Roelants I, Rurangwa E, Moore HDM, Ollevier F (1996). Use of computer assisted sperm analysis (CASA) for monitoring
sperm quality of fish; application for effects of heavy
metals. Aquat Toxicol.

[r22] Koskimies AI, Savander M, Ann-Marie N, Kurunmäki H (2010). Sperm DNA damage and male infertility. Duodecim.

[r23] Marchlewicz M, Wiszniewska B, Gonet B, Baranowska-Bosiacka I, Safranow K, Kolasa A, Głabowski W, Kurzawa R, Jakubowska K, Rać ME (2007). Increased lipid peroxidation and ascorbic Acid utilization in
testis and epididymis of rats chronically exposed to lead. Biometals.

[r24] Meligy AMA, Waheed MM, El-Bahr SM (2019). Effect of heavy metals arsenic, cadmium, and lead on the semen
variables of dromedary camels (Camelus dromedarius). Anim Reprod Sci.

[r25] Omari MO, Kibet JK, Cherutoi JK, Bosire JO, Rono NK (2015). Heavy metal content in mainstream cigarette smoke of common
cigarettes sold in Kenya, and their toxicological
consequences. Int Res J Environ Sci.

[r26] Omu AE, Dashti H, Mohamed AT, Mattappallil AB (1995). Significance of trace elements in seminal plasma of infertile
men. Nutrition.

[r27] Pacey AA, Cosson JC, Bentley MG (1994). Intermittent swimming in the spermatozoa of the lugworm Arenicola
marina (L.) (Annelida: Polychaeta). Cell Motil Cytoskeleton.

[r28] Parvizi N, Ellendorff F (1982). Further evidence on dual effects of norepinephrine on LH
secretion. Neuroendocrinology.

[r29] Písa J, Cibulka J, Ptácek M (1990). Effect of subcutaneous application of a single cadmium dose on
oocyte maturation in vitro. Physiol Bohemoslov.

[r30] Preston BL, Snell TW (2001). Full life-cycle toxicity assessment using rotifer resting egg
production: implications for ecological risk assessment. Environ Pollut.

[r31] Saksena SK, Dahlgren L, Lau IF, Chang MC (1977). Reproductive and endocrinological features of male rats after
treatment with cadmium chloride. Biol Reprod.

[r32] Sarvari A, Naderi MM, Heidari M, Zarnani AH, Jeddi-Tehrani M, Sadeghi MR, Akhondi MM (2010). Effect of Environmental Risk Factors on Human
Fertility. J Reprod Infertil.

[r33] Shafi H, Agajani Delavar M, Esmaeilzadeh S (2016). Comparing the Prevalence of Infertility in Urban and Rural Areas
in Babol. J Mazandaran Univ Med Sci.

[r34] Sharma R, Panwar K, Barber I, Purohit A (2013). Lead toxicity and postnatal development of ovary. Int J Pharm Sci Res.

[r35] Shimbo S, Zhang ZW, Watanabe T, Nakatsuka H, Matsuda-Inoguchi N, Higashikawa K, Ikeda M (2001). Cadmium and lead contents in rice and other cereal products in
Japan in 1998-2000. Sci Total Environ.

[r36] Simon L, Proutski I, Stevenson M, Jennings D, McManus J, Lutton D, Lewis SE (2013). Sperm DNA damage has a negative association with live-birth rates
after IVF. Reprod Biomed Online.

[r37] Sokol RZ. (1987). Hormonal effects of lead acetate in the male rat: mechanism of
action. Biol Reprod.

[r38] Teijón C, Olmo R, Blanco D, Romero A, Teijón JM (2006). Low doses of lead: effects on reproduction and development in
rats. Biol Trace Elem Res.

[r39] Wegner CC, Clifford AL, Jilbert PM, Henry MA, Gentry WL (2010). Abnormally high body mass index and tobacco use are associated
with poor sperm quality as revealed by reduced sperm binding to
hyaluronan-coated slides. Fertil Steril.

[r40] Zarif Golbar Yazdi H, Aghamohammadian Sharbaf H, Kareshki H, Amirian M (2020). Infertility and Psychological and Social Health of Iranian
Infertile Women: A Systematic Review. Iran J Psychiatry.

